# Diverse accumulation of several dehydrin-like proteins in cauliflower (*Brassica oleracea *var. *botrytis*), *Arabidopsis thaliana *and yellow lupin (*Lupinus luteus*) mitochondria under cold and heat stress

**DOI:** 10.1186/1471-2229-10-181

**Published:** 2010-08-18

**Authors:** Michal Rurek

**Affiliations:** 1 Department of Molecular and Cellular Biology, Institute of Molecular Biology and Biotechnology, Adam Mickiewicz University, Umultowska 89, 61-614 Poznan, Poland

## Abstract

**Background:**

Dehydrins represent hydrophilic proteins acting mainly during cell dehydration and stress response. Dehydrins are generally thermostable; however, the so-called dehydrin-like (dehydrin-related) proteins show variable thermolability. Both groups immunoreact with antibodies directed against the K-segment of dehydrins. Plant mitochondrial dehydrin-like proteins are poorly characterized. The purpose of this study was to extend previous reports on plant dehydrins by comparing the level of immunoprecipitated dehydrin-like proteins in cauliflower (*Brassica oleracea *var. *botrytis*), *Arabidopsis thaliana *and yellow lupin (*Lupinus luteus*) mitochondria under cold and heat stress.

**Results:**

All the analyzed plant species showed constitutive accumulation of thermostable mitochondrial putative dehydrins ranging from 50 to 70 kDa. The mitochondrial dehydrin-like proteins observed in cauliflower and *Arabidopsis *ranged from 10 to 100 kDa and in lupin imbibed seeds and hypocotyls - from 20 to 90 kDa. Cold treatment increased mainly the accumulation of 10-100 kDa cauliflower and *Arabidopsis *dehydrin-like proteins, in the patterns different in cauliflower leaf and inflorescence mitochondria. However, in lupin mitochondria, cold affected mainly 25-50 kDa proteins and seemed to induce the appearance of some novel dehydrin-like proteins. The influence of frost stress on cauliflower leaf mitochondrial dehydrin- like proteins was less significant. The impact of heat stress was less significant in lupin and *Arabidopsis *than in cauliflower inflorescence mitochondria. Cauliflower mitochondrial dehydrin-like proteins are localized mostly in the mitochondrial matrix; it seems that some of them may interact with mitochondrial membranes.

**Conclusions:**

All the results reveal an unexpectedly broad spectrum of dehydrin-like proteins accumulated during some abiotic stress in the mitochondria of the plant species analyzed. They display only limited similarity in size to those reported previously in maize, wheat and rye mitochondria. Some small thermolabile dehydrin-like proteins were induced under stress conditions applied and therefore they are likely to be involved in stress response.

## Background

Stress acclimation in plants is associated with the appearance of characteristic cellular, biochemical and gene expression alterations [1]. It is assumed that the LEA protein family is engaged, among other functions, in the process of cellular response to stress conditions. LEA proteins show significant evolutionary conservation [2,3]. The second group of LEA family, represented by dehydrins, contains hydrophilic, lysine- and glycine-rich proteins accumulating during seed germination and maturation. Some dehydrins are inducible under different stress conditions such as drought, low temperature, freezing, salinity and ABA treatment [4-7]. Dehydrins share the segmental structure, which makes it possible to divide them into five subclasses. All dehydrins contain the lysine-rich conserved domain named the K-segment, characterized by the 15- amino acid consensus motif EKKGIMDKIKEKLPG [4,8]. It is generally assumed that dehydrins protect the rapidly growing plant organs against damage. They are present both in the nucleus and in the cytoplasm and also close to the elements of cytoskeleton as well as in the vicinity of the plasma membrane and tonoplast [9-13]. The data concerning potential associations of dehydrins with other cell compartments are still limited and need more verification.

A few members of the dehydrin group were clearly shown to be heat-stable (e.g. the peach PCA60 protein [14]) and resistant to structural collapse (e.g. three *Arabidopsis *dehydrins [15]). However, numerous related proteins - known as 'dehydrin-like proteins' (dlps) or 'dehydrin-related proteins' - quite often display variable levels of thermostability [16]. The data on their full-size cDNAs or genomic sequences as well as results of functional analyses are very often unavailable. 'Dehydrin-like' or 'dehydrin-related proteins' crossreact with K-segment specific antibodies, which indicates that they may contain a key aminoacid motif- K-segment, similar to the one found in dehydrins, with some extent of degeneration. In general, 'dehydrin-like proteins', immunologically related to dehydrins, are a poorly characterized group of proteins and they need to be explored thoroughly. Nowadays, some of them may be classified as 'genuine dehydrins' rather than as dlps if they share all the properties typical of dehydrins.

Data concerning the occurrence of dehydrins and proteins related to dehydrins in higher plant mitochondria are very scarce. Recently, using the method of immunogold tissue staining with antibodies against the K-segment of dehydrin, it was shown that constitutive dlps, which associate with the mitochondria of *Chenopodium *embryonic axes and cotyledons, are active in the seed development program [17]. Moreover, the association of *Citrus unshiu *cold-responsive COR19 dehydrin - overexpressed in *Nicotiana *- was observed in the fraction enriched in crude mitochondria [18-20]. The results of localisation of dehydrin-related proteins contradicted the other, earlier data obtained from tissue immunogold staining and Western blot analyses of cellular fractions enriched in mitochondria of other plants [7,9]. Although *Arabidopsis *expression products of ten known dehydrin genes ([Gen Bank:NP_173468, NP_850947, NP_175843, NP_177745, NP_179744, NP_190666, NP_190667, NP_195554, NP_195624, NP_201441]) are not predicted to be targeted to mitochondria [2], some other plant LEA proteins could be regarded as putative mitochondrial proteins [21]. This may be consistent with the recent discovery of a cloned, non-dehydrin, organellar LEA III family member that is targeted to the pea mitochondria matrix (shown by functional tests; [21,22]). Interestingly, LEA III family members characterized in wheat and rye chloroplasts displayed elevated accumulation under cold stress [23].

The participation of 'true' mitochondrial dehydrin-like proteins in stress response under cold, drought, frost and ABA treatment was confirmed only recently in cereals. Initially, in winter wheat, winter rye and maize mitochondria, two dlps - of ca. 50 and 60 kDa - were identified [24]. The authors of this paper also showed that during cold stress response, mostly in wheat, the accumulation of the 50 kDa dlp was increased; however, in rye mitochondria this effect concerned 60 kDa protein. It was also proved that the dlps of 50/54 and 60 kDa which accumulated in wheat and rye mitochondria were highly thermostable [25]. The same authors also provided evidence for a positive correlation between the levels of these proteins in rye and wheat mitochondria in response to cold stress and cold acclimation. They suggested that during cold or osmotic stress mitochondrial dlps were involved either in the cellular response against freezing/low temperature or in the regulation of mitochondrial functions [25]. Besides high-molecular weight dlps, the presence of thermolabile lower- molecular weight dlps in the mitochondrial extracts of winter wheat, rye and maize grown under control conditions was also documented [16]. The authors [16] showed that the wheat and rye proteins of 52 kDa and 63 kDa (recalculation of earlier estimated size of 50 kDa and 60 kDa proteins by Borovskii *et al. *[24]) were highly accumulated during the diverse stress conditions; however, the wheat, rye and maize thermolabile dlps of molecular weight 28 and 56-59 kDa were accumulated in a rather constitutive manner under the same conditions. The influence of ABA on thermostable dlps was the highest in rye and the lowest in maize mitochondria [16]. The same authors characterized the interactions of cytoplasmic dehydrins with mitochondria during cold treatment of winter wheat plants. According to their results, the cold-induced winter wheat dehydrin, which displayed an elevated level in cytoplasm after acclimation to cold stress, associated rapidly with the mitochondrial outer membrane [26].

The aim of the present work was to better understand the function of dlps in plant mitochondria by extending the above - described studies over three different plant species which had not been analyzed previously. Comparative analysis of the levels of dlps in cauliflower, *Arabidopsis *and yellow lupin mitochondria in response to cold, freeze and heat stress was performed. Also, some results concerning the association of cauliflower dlps with the fraction enriched in membranes are presented here. Moreover, the influence of the analyzed stress conditions on the appearance of dlps in different plant organs was investigated.

## Results

### Accumulation of dehydrin-like proteins in cauliflower and *Arabidopsis *mitochondria

**Figure 1:**
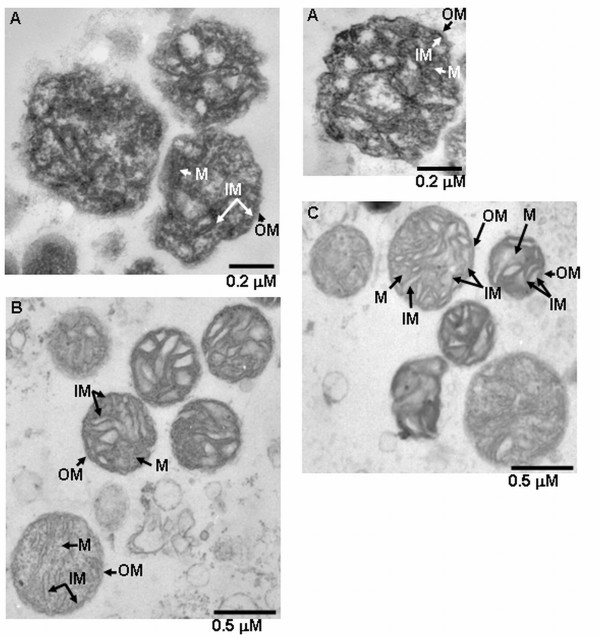
**Trasmission electron micrographs of mitochondria from cauliflower and yellow lupin**. The quality of mitochondria isolated from cauliflower inflorescences (**A**), yellow lupin imbibed seeds (**B**) as well as from yellow lupin hypocotyls (**C**) was analyzed using transmission electron microscope. In the images, outer (**OM**), inner mitochondrial membrane (**IM**), and mitochondrial matrix (**M**) are indicated (**A **bar = 0.2 μm; **B, C **bars = 0.5 μm).

**Figure 2:**
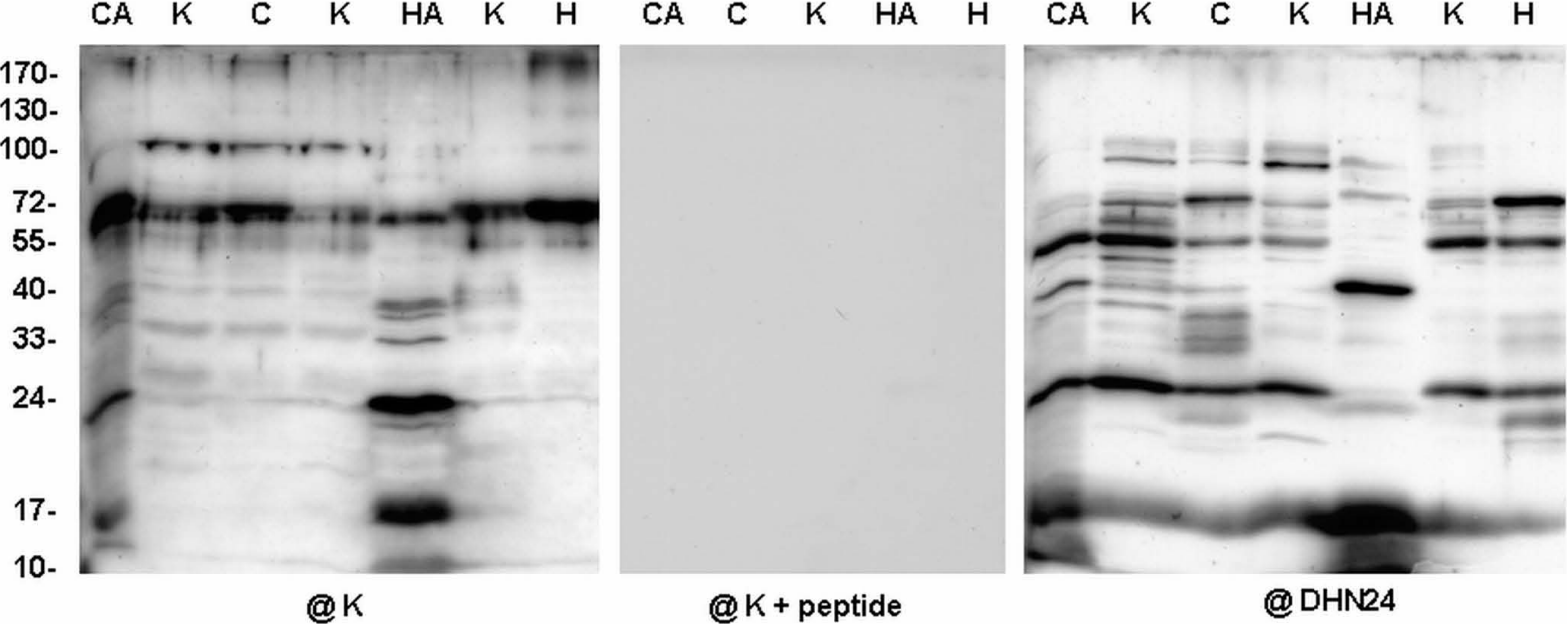
**Western analysis of dehydrin-like proteins in the mitochondria from cauliflower inflorescences**. Cauliflower plants were grown under control conditions (**K**), subjected to heat (**H**) or cold (**C**) stress, or grown under standard growth conditions after stopping the heat (**HA**) or cold (**CA**) treatment. Ten micrograms of mitochondrial proteins were loaded per lane. Western analysis was carried out using the antibody against the K-segment of dehydrins (**@K**), the same antibody blocked with peptide (**@K + peptide**) or the antibody against the DHN24 dehydrin (**@DHN24**). The representative Western blots came from 3 experimental repetitions.

**Figure 3:**
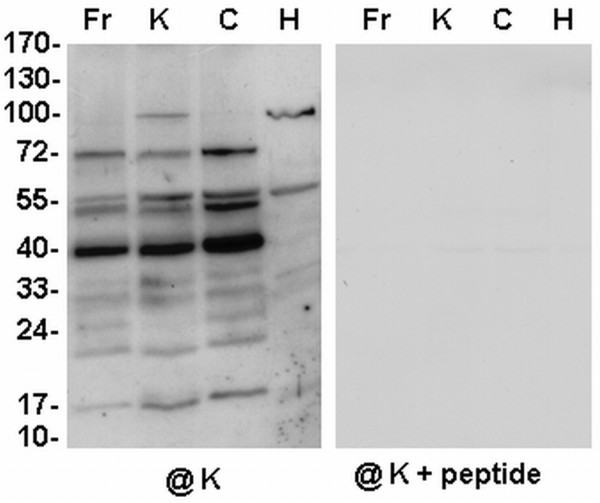
**Western analysis of dehydrin-like proteins in the mitochondria from cauliflower leaves**. Cauliflower plants were grown in control conditions (**K**), or they were subjected to a short freeze (**Fr**), cold (**C**) or heat (**H**) stress. Ten micrograms of mitochondrial proteins were loaded per lane. Western analysis was carried out using the antibody against K-segment of dehydrins (**@K**) and the same antibody blocked with peptide (**@K + peptide**). The representative Western blots came from 3 experimental repetitions.

To analyse accumulation of dehydrin-like proteins in cauliflower mitochondria under control and stress conditions, the mitochondria from leaves as well as from young inflorescences were isolated and their purity was assayed by transmission electron microscopy (Figure 1A). Moreover, no protein was detected on any immunoblot when the K- segment specific antibodies were blocked with the synthetic peptide for this segment confirming the specificity of antibody recognition (Figures 2, 3).

The mitochondria obtained from cauliflower inflorescences of plants grown under standard conditions showed similar pattern of dlps immunoreacting with K-segment specific antibodies (one from Stressgen and the other described by Close *et al. *[27], Figure 2). The highly abundant dlps ranged from ca. 55 to 100 kDa. However, with the antibody against SK_3 _segments of *Solanum sogarandinum *DHN24 dehydrin [6], strong accumulation of ca. 15, 25, 55 and 100 kDa dehydrin-related proteins was noticed (Figure 2, Table 1). Under cold and heat stress an increased level of K-segment immunoreactive dlps of ca. 50-70 kDa was visible; however, the profile of smaller proteins was different after the above treatments (Figure 2). When the antibody against the DHN24 dehydrin was used, the changes in the content of dlps under the same stress treatment were not similar to those obtained with K-segment specific antibodies (Table 1). Additionally, all the used antibodies permitted detection of more evidently accumulated dlps of ca. 10, 15, 25, 40 and 55-65 kDa in mitochondria of cauliflower plants recovered after stress treatment (Figure 2, Table 1).

**Table 1:** Changes in the relative level of dlps in cauliflower mitochondria.

Cauliflower (*Brassica oleracea *var. *botrytis*)						
**Antibody**	**K**					

**Organ**	**Leaves**					

**Stress** **treatment**	**Size (kDa)**	**Control**	**Cold**	**Freeze**	**Heat**	

	15	26.9	53.0	15.6	n.d.	
	
	20	25.9	42.9	24.8	n.d.	
	
	25	n.d.	42.7	27.3	n.d.	
	
	30	27.9	56.9	38.3	63.3	
	
	35	20.5	51.6	27.5	56.9	
	
	40	78.1	143.3	101.8	40.3	
	
	50	60.7	138.7	68.3	n.d.	
	
	55	76.7	89.2	42.1	69.0	
	
	70	53.8	104.3	61.4	n.d.	
	
	100	19.4	n.d.	n.d.	131.1	

**Antibody**	**K**					

**Organ**	**Early inflorescences**					

**Stress** **treatment**	**Size (kDa)**	**Control**	**Cold**	**Cold +** **control growth**	**Heat**	**Heat +** **control** **growth**

	10	7.4	4.5	61.0	9.5	109.2
	
	15	20.4	12.3	102.5	3.1	177.9
	
	25	41.4	37.5	121.6	15.0	190.8
	
	35	34.8	49.7	59.2	2.2	57.1
	
	40	30.3	38.8	91.7	2.0	91.4
	
	**50**	57.6	76.8	79.6	42.7	10.8
	
	**55**	89.9	118.0	120.3	113.4	33.4
	
	**65**	141.8	187.3	153.9	197.0	149.3
	
	**70**	76.6	153.3	142.3	197.3	58.6
	
	**100**	91.0	116.3	38.4	47.9	6.2

**Antibody**	**DHN24**					

**Organ**	**Early inflorescences**					

**Stress** **treatment**	**Size (kDa)**	**Control**	**Cold**	**Cold +** **control** **growth**	**Heat**	**Heat +** **control growth**

	10	83.6	11.0	152.6	n.d.	202.5
	
	15	116.7	118.2	194.0	89.2	221.8
	
	25	129.1	128.9	163.2	110.8	17.6
	
	30	16.8	101.1	48.0	23.4	6.5
	
	35	18.6	93.2	57.6	43.3	13.5
	
	40	14.7	27.4	96.3	23.3	136.2
	
	**50**	8.4	9.0	48.6	19.6	14.0
	
	**55**	62.3	68.7	158.5	108.9	4.7
	
	**65**	36.3	41.4	59.7	63.5	6.2
	
	**70**	37.7	91.6	44.6	134.1	27.9

Thermostable proteins are denoted in bold. Thermolabile proteins (considered as not resistant for boiling in 15 min.) are underlined. **n.d**.- not detected or a very low-abundant protein. The thermostability of mitochondrial dlps from leaves of cauliflower was not determined. For the isolation of mitochondria, the cauliflower inflorescences were taken either directly after stress treatment (**cold**, **heat**) or after the subsequent plant growth under control conditions (**cold + control growth**, **heat + control growth**). The results of densitometrical analysis of three independent Western blots were presented as average value in arbitrary units after background subtraction, as the optical density of protein bands per square pixel.

In the cauliflower leaves the yield of pure mitochondria was significantly lower than in cauliflower inflorescences. Cold and heat plant treatment as well as short plant freezing were used for the analysis of cauliflower leaf mitochondrial dlps under stress conditions (Figure 3). In the mitochondria from cauliflower leaves grown under control conditions, K-segment specific dlps different from those in inflorescences were accumulated, for instance ca. 40, 50, 55 and 70 kDa (Figures 2, 3, Table 1). Cold stress plant treatment, contrary to freezing, resulted mainly in increase in the dlps of ca. 15 kDa and larger than 50 kDa. However, heat stress resulted in extensive accumulation mainly of ca. 100 kDa dlp (Figure 3). The application of the antibody against the DHN24 dehydrin revealed some changes in the abundance of dlps from the mitochondria of leaves during all stress treatments, although it did not distinguish well between the cold and freeze induced dlps (data not shown).

**Figure 4:**
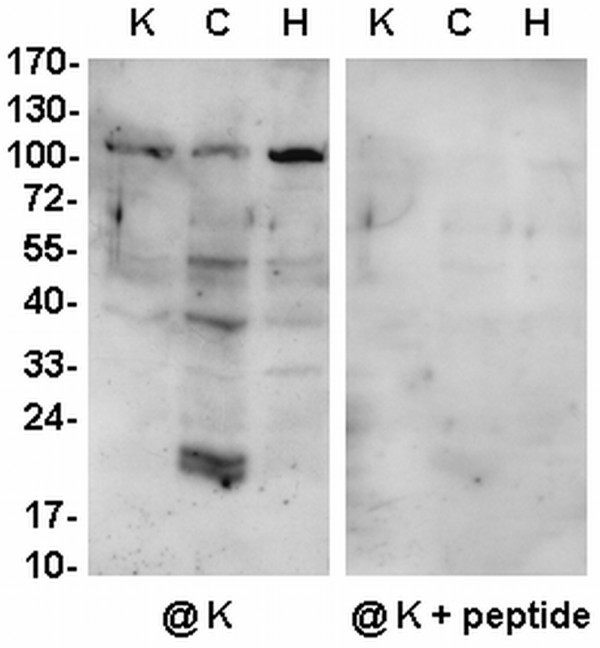
**Western analysis of dehydrin-like proteins in the mitochondria from *Arabidopsis *leaves**. *Arabidopsis *plants were grown under control conditions (**K**), or subjected to cold (**C**) or heat (**H**) stress. Ten micrograms of mitochondrial proteins were loaded per lane. Western analysis was carried out using the antibody against the K-segment of dehydrins (**@K**). The representative Western blots came from 3 experimental repetitions.

**Figure 5:**
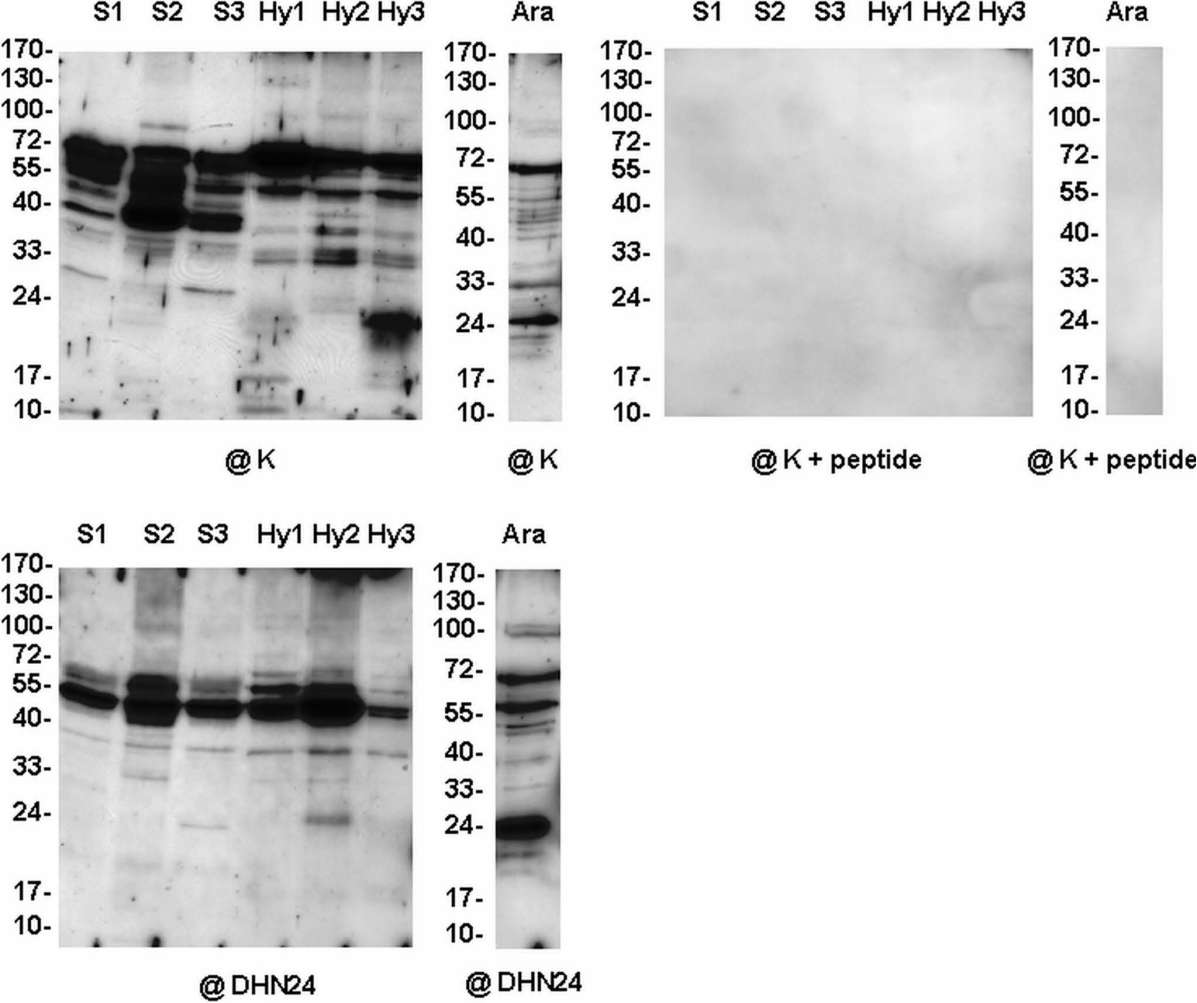
**Western analysis of dehydrin-like proteins in yellow lupin mitochondria**. Dlps were analyzed in mitochondrial extracts from imbibed seeds (**S**) and seedling hypocotyls (**Hy**) of yellow lupin and from the mitochondria of *Arabidopsis *cell cultures (**Ara**). The lupin imbibed seeds and seedlings hypocotyls were grown under control conditions (**1**), or subjected to cold (**2**) or heat (**3**) treatment. Ten micrograms of mitochondrial proteins were loaded per lane. Western analysis was carried out using the antibody against K-segment of dehydrins (**@K**), the same antibody blocked with peptide (**@K + peptide**) and the antibody against DHN24 dehydrin (**@DHN24**). The representative Western blots came from 3 experimental repetitions.

The mitochondria from *Arabidopsis *were prepared from mature leaves and 6-day-old cell cultures (Figures 4, 5, again with lower yield comparing to the yield from cauliflower inflorescences. In rosette leaf mitochondria of *Arabidopsis *plants grown under control conditions, antibodies against the K-segment of dehydrins permitted detection of only a few dlps (ca. 40 to 100 kDa). No protein was found on any immunoblot when the K-segment specific antibodies were blocked with the synthetic peptide for this segment (Figure 4). Cold treatment of *Arabidopsis *plants caused evident induction of ca. 20 kDa dlp, and a significant increase in the amount of the remaining dlps (especially ca. 40 and 55 kDa). The impact of heat stress resulted in elevated accumulation of ca. 100 kDa dlp (Figure 4, Table 2). Immunoassay with DHN24 antibodies did not reveal any qualitative changes (data not shown).

**Table 2:** Changes in the relative level of dlps in *Arabidopsis *mitochondria.

*Arabidopsis thaliana*								
**Antibody**	**K**						**DHN24**	

**Organ**	**Rosette leaves**				**Cell cultures**			

**Stress treatment**	**Size (kDa)**	**Control**	**Cold**	**Heat**	**Size (kDa)**	**Control**	**Size (kDa)**	**Control**

	20	n.d.	89.0	n.d.	20	57.4	20	68.5
	
	40	20.0	67.0	26.4	25	122.2	25	137.5
	
	50	36.1	53.2	27.1	35	72.4	40	29.7
	
	55	39.2	66.4	30.8	45	65.0	**50**	66.6
	
	100	86.6	58.4	108.9	**50**	65.2	**65**	110.3
	
					**55**	65.7	**70**	114.3
					
					**65**	56.7	100	46.3
					
					**70**	133.1		

Thermostable proteins are denoted in bold. Thermolabile proteins (considered as not resistant for boiling in 15 min.) are underlined. **n.d**.- not detected or a very low-abundant protein. The thermostability of mitochondrial dlps from leaves of *Arabidopsis *was not determined. The results of densitometrical analysis of three independent Western blots were presented as average value in arbitrary units after background subtraction, as the optical density of protein bands per square pixel.

The pattern of dlps in the mitochondria isolated from *Arabidopsis *cell cultures differed from that of *Arabidopsis *leaves (Figure 5, Table 2). The highly accumulated dlps detected with antibodies against the K-segment of dehydrins were the proteins of ca. 25 and 70 kDa and with DHN24 antibody - of ca. 25 and 50-70 kDa.

### Accumulation of dehydrin-like proteins in the mitochondria of imbibed seeds and hypocotyl of yellow lupin in response to cold and heat stress

Mitochondria from both lupin imbibed seeds and 6-day-old-hypocotyls were used to analyse the accumulation of dehydrin-like proteins in yellow lupin mitochondria under control and stress conditions. The high purity of lupin organelles and the integrity of their membranes was confirmed by microscopic analyses (Figure 1B-C). Mitochondrial dlps from yellow lupin imbibed seeds and hypocotyls were analyzed with two antibodies raised against the dehydrin K-segment as well as with the antibody against DHN24 dehydrin. No protein was detected on any immunoblot when the K-segment specific antibody [27] was blocked with the synthetic peptide for this segment (Figure 5).

In lupin imbibed seeds and hypocotyl mitochondria grown under control conditions a few distinct dlps were detected with all antibodies, with the most abundant proteins of ca. 40-70 kDa (Figure 5, Table 3). Additionally, a few smaller dlps of ca. 20-40 kDa immunoreacted with antibodies against the K-segment of dehydrins. Interestingly, in the mitochondria of lupin imbibed seeds the low-molecular weight dlps (below 30 kDa) were generally less abundant than in those obtained from hypocotyls (Figure 5). The sensitivity of detection of the mitochondrial small dlps in different organs of lupin with the antibody for DHN24 dehydrin, was quite low (Figure 5).

**Table 3:** Changes in the relative level of dlps in yellow lupin mitochondria.

Yellow lupin (*Lupinus luteus*)								
**Antibody**	**K**							

**Organ**	**Imbibed seeds**				**Hypocotyls**			

**Stress** **treatment**	**Size (kDa)**	**Control**	**Cold**	**Heat**	**Size (kDa)**	**Control**	**Cold**	**Heat**

	30	35.0	29.2	53.7	20	44.3	n.d.	115.0
	
	35	42.4	107.3	62.5	25	44.2	34.5	160.5
	
	40	63.3	177.0	131.5	35	54.3	120.3	85.0
	
	45	102.1	188.8	97.7	40	31.8	83.0	53.5
	
	55	171.5	183.5	144.3	45	n.d.	44.3	n.d.
	
	65/70	171.6	163.2	180.0	**50/55**	144.7	120.5	131.0
	
	80	n.d.	30.3	n.d.	**65**	187.5	123.0	133.3
	
					**70**	175.0	179.4	179.0

**Antibody**	**DHN24**							

**Organ**	**Imbibed seeds**				**Hypocotyls**			

**Stress** **treatment**	**Size (kDa)**	**Control**	**Cold**	**Heat**	**Size (kDa)**	**Control**	**Cold**	**Heat**

	35	n.d.	18.3	n.d.	25	n.d.	43.1	n.d.
	
	40	12.5	27.2	16.8	40	25.3	47.4	n.d.
	
	50	40.6	108.9	39.4	**50**	121.9	148.8	79.0
	
	55	136.8	136.1	138.2	**55**	146.0	151.0	86.6
	
	65	87.6	123.7	78.9	**65**	117.8	132.3	39.7
	
	90	n.d.	34.1	10.3	**70**	41.7	58.0	23.4

Thermostable proteins are denoted in bold. Thermolabile proteins (considered as not resistant for boiling in 15 min.) are underlined. **n.d**.- not detected or a very low- abundant protein. The thermostability of dlps of the lupin imbibed seed mitochondria was not determined. The results of densitometrical analysis of three independent Western blots were presented as average value in arbitrary units after background subtraction, as the optical density of protein bands per square pixel.

The influence of cold and heat stress on the appearance of dlps in the mitochondria of yellow lupin isolated from different organs was different. Immunoanalyses with K-segment specific antibodies revealed that cold stress up-regulates the accumulation of small dlps (ranging from ca. 35-45 kDa) in the mitochondria from lupin hypocotyls and especially from imbibed seeds. However, the level of ca. 55 and 65 kDa dlps remained stable (Figure 5). By means of the antibody against DHN24 dehydrin for the immunodetection of dlps under cold stress, the induction of accumulation of ca. 25 kDa protein and an increase in the level of ca. 40 kDa dlp were observed in the hypocotyl mitochondria. In the imbibed seed mitochondria, cold treatment resulted in up-regulation of dlps from 40-90 kDa (Figure 5, Table 3). Heat stress caused the up-regulation of 20-40 kDa K-segment immunoreactive dlps in hypocotyl mitochondria (Figure 5). In the lupin imbibed seed mitochondria heat stress increased mainly the level of the dlps of ca. 30 and 40 kDa. The antibody against the DHN24 dehydrin under heat stress helped detect only minor changes in the level of mitochondrial dlps from yellow lupin imbibed seeds.

### Subcellular localisation of cauliflower dehydrin-like proteins

**Figure 6:**
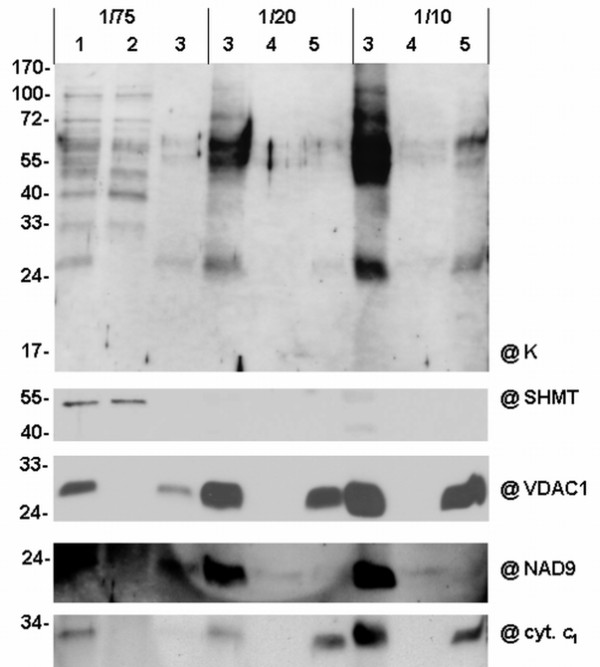
**Western analysis of dehydrin- like proteins in cauliflower mitochondrial subfractions**. Mitochondria from cauliflower inflorescences (**1**) were fractionated into fractions enriched in soluble matrix proteins (**2**), membrane proteins (**3**), peripheral membrane proteins (**4**) and in integral membrane proteins (**5**). Equal amounts of each subfraction volume were loaded onto the gel (ca. 1/75, 1/20 and 1/10). Peripheral and integral membrane proteins were loaded after acetone precipitation (lanes **4 **and **5**). Western analysis was carried out using the antibody against the K-segment of dehydrins (**@K**). Control antibodies directed against SHMT, NAD9 subunit of complex I, cyt. *c*_1 _and VDAC-1 for the monitoring of purity of fractions were additionally used (panels below). The representative Western blots came from 3 experimental repetitions.

For the analysis of the intramitochondrial localisation of dlps, mitochondria were isolated from cauliflower inflorescences, hypotonically lyzed, sonicated and fractionated into submitochondrial fractions. Equal parts (by volume) of each fraction were analyzed on Western blots with antibodies specific to the K-segment of dehydrins. In order to check the quality and determine the purity of the obtained submitochondrial fractions, Western blot analyses with antibodies against control proteins localized specifically within mitochondria were performed. Those analyses confirmed good quality of the obtained fractions, because NAD9 subunit of mitochondrial complex I was localized in the peripheral membrane fraction, contrary to cyt. *c*_1 _and VDAC-1, which were abundant in the integral membrane fraction. SHMT and IDH were localized in the fraction enriched in the matrix proteins (Figure 6).

Most of the putative cauliflower mitochondrial dlps seemed to be localized in the matrix (the thermolabile dlps of ca. 35 and 40, and the thermostable proteins of ca. 50, 60, 70 and 100 kDa); however, there were three proteins present in the fraction enriched in mitochondrial membranes: the thermostable dlps (ca. 55, 65 kDa) and the thermolabile one (ca. 25 kDa, Figure 6). To obtain more data on the localisation of membrane associated dlps, alkali treatment of the cauliflower mitochondrial membranes and subsequent separation into the peripheral and the integral protein subfractions were performed. The thermolabile dlps of ca. 25 kDa as well as the thermostable dlps of ca. 55 and 65 kDa were found mostly in the integral protein subfraction (Figure 6).

### Interactions of cauliflower dehydrin-like proteins with the mitochondrial fraction enriched in membranes

In order to study in more detail the interaction of cauliflower dlps with mitochondrial membranes, additional assays were carried out. One concerned potassium chloride extraction from all proteins loosely attached to mitochondrial membranes, and the other included proteolytic digestion of cauliflower intact mitochondria - with a view to investigate special interactions of dlps with the outer mitochondrial membrane. In order to obtain accurate results, salt extraction was conducted twice: with or without sonication, as this step could have resulted in an artifactual release of dlps from mitochondrial membranes to the soluble protein fraction. As control experiments, mitochondrial extracts were treated with equal volumes of phosphate buffer instead of KCl.

**Figure 7:**
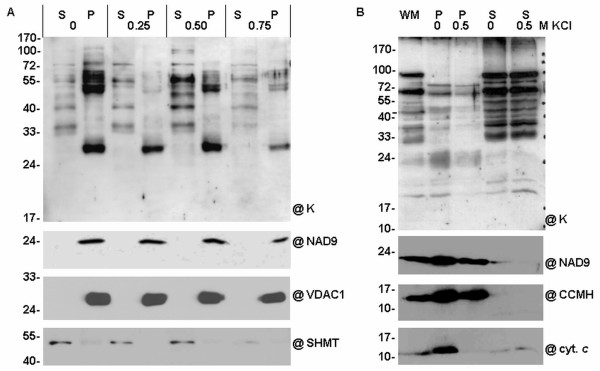
**Extraction of cauliflower mitochondrial dehydrin-like proteins by salt treatment**. Dlps were extracted by KCl treatment in the case of unsonicated (**A**) and sonicated (**B**) cauliflower mitochondria. Cauliflower mitochondria (**WM**) were hypotonically lyzed and after the subsequent sonication (**B**) or without sonication (**A**) they were submitted to protein extraction at different concentrations of KCl (0, 0.25, 0.50, 0.75 M). After that all the samples were centrifuged and the supernatants (**S**) containing soluble proteins and pellets (**P**) enriched in membrane proteins were gel analyzed. Equal protein amounts by volume were set per lane (1/75). Western analysis was carried out using the antibody against the K-segment of dehydrins (**@K**). Control antibodies directed against SHMT, NAD9 subunit of complex I, CCMH protein, cyt. *c *and VDAC-1 for the monitoring of the purity of fractions were additionally used (panels below). The representative Western blots came from 3 experimental repetitions.

Salt treatment was performed using extracts of gently lyzed cauliflower mitochondria (not submitted to sonication). After completion of the salt extraction, mitochondrial lysate was ultracentrifuged and fractions enriched in mitochondrial membranes as well as a supernatant enriched in soluble and mebrane-stripped proteins were used for further analyses. For the assessment of the quality of KCl extraction of peripheral membrane proteins without the sonication step, the control antibodies directed against SHMT, subunit NAD9 of complex I, the cyt. *c*_1 _and VDAC-1 were used. The control immunoassays confirmed good quality of the fractions prepared, because SHMT was localized exclusively in the soluble fraction, whereas the remaining fractionation markers were in the fractions enriched in membranes (Figure 7A). In order to remove peripheral proteins efficiently, the optimal concentration of KCl was determined. Antibodies against the K-segment of dehydrins made it possible to determine the most effective KCl concentration in dlps extraction as 0.5 M. At 0.5 M KCl the dlps of ca. 50-65 kDa were partially stripped from fractions enriched in membranes. Moreover, at 0.75 M salt concentration most of those thermostable dlps were removed from the mitochondrial membranes (Figure 7A).

A salt treatment was also performed with sonicated mitochondria (Figure 7B). The quality of KCl extraction of peripheral membrane proteins with the sonication step was also tested with control antibodies. They were directed against CCMH protein, NAD9 subunit of complex I and cyt. *c*. The control immunoassays showed that sonication and high concentration salt treatment did not significantly remove proteins containing membrane domains, like CCMH, or proteins in complexes, like NAD9 (Figure 7B). However, at 0.5 M KCl a significant release of cyt. *c *from the membrane fraction was observed, due to the fact that this protein was loosely attached to the inner mitochondrial membrane. The analysis of dlps immunoreactive with K-segment specific antibodies confirmed that some dlps (for instance, ca. 25 kDa) were removed at 0.5 M KCl (Figure 7B). However, a dlp of ca. 65 kDa was found in soluble fractions even without salt treatment, which may indicate that this protein had been stripped off the mitochondrial membranes during sonication. Generally, cauliflower mitochondria contain a set of membrane-associated dlps that may be released during the treatment of mitochondria with high salt concentrations.

**Figure 8:**
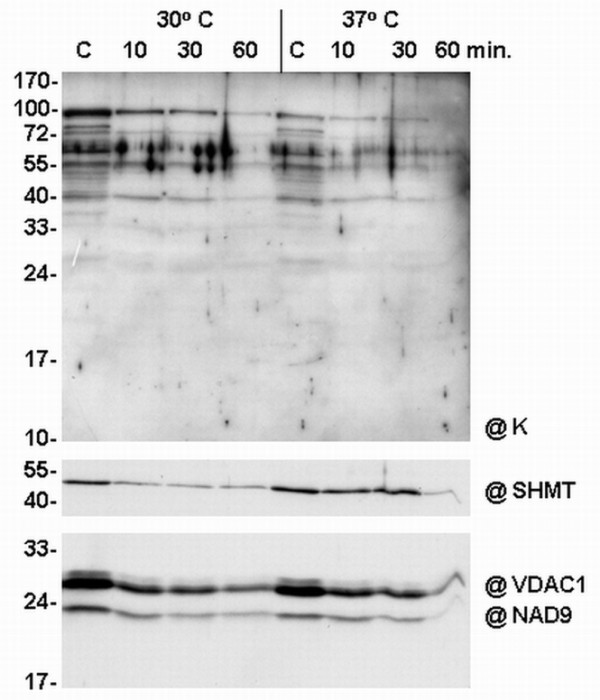
**Analysis of dehydrin-like proteins during pronase treatment of cauliflower mitochondria**. The analysis was conducted at two temperatures (30°C and 37°C) and at three time points (10, 30 and 60 min.). **C**- control: cauliflower mitochondria untreated with pronase. Thirty micrograms of mitochondrial proteins were set per lane. Western analysis was carried out using the antibody against the K-segment of dehydrins (**@K**). Control antibodies directed against SHMT, VDAC-1 and the NAD9 subunit of complex I for the monitoring of the quality of cauliflower mitochondria during proteolytic digestion were additionally used (panels below). The representative Western blots came from 3 experimental repetitions.

The intactness of cauliflower mitochondria during pronase E digestion was confirmed using antibodies against SHMT, VDAC-1 and NAD9 subunit of mitochondrial complex I (Figure 8). As the proteolytic digestion of certain membrane proteins had to be studied more carefully, the digestion of freshly isolated cauliflower mitochondria proceeded at two temperatures and for three time intervals (Figure 8). The incubation temperature of 30°C and the digestion time intervals up to 30 min were found to be optimal. According to the results of pronase E digestion, only membrane-bound dlps that immunoreacted with K-segment specific antibodies were sensitive to proteolytic digestion. The thermostable dlp of ca. 65 kDa, which could be removed from membranes by salt treatment (Figure 7A) seems to be a putative candidate for a dehydrin interacting with outer mitochondria (probably from the cytoplasmic side), because its proteolysis proceeded in a short time even at 30°C (Figure 8). However, another thermostable protein - the dlp of ca. 55 kDa - was more resistant to the proteolytic action of pronase E, which indicated that it could be associated either with the inner mitochondrial membrane or with the outer mitochondrial membrane from the intermembrane space. The resistance to pronase E digestion was also observed for all the putative matrix-located dlps (Figure 8).

### Analysis of thermostability of chosen dehydrin-like proteins

**Figure 9:**
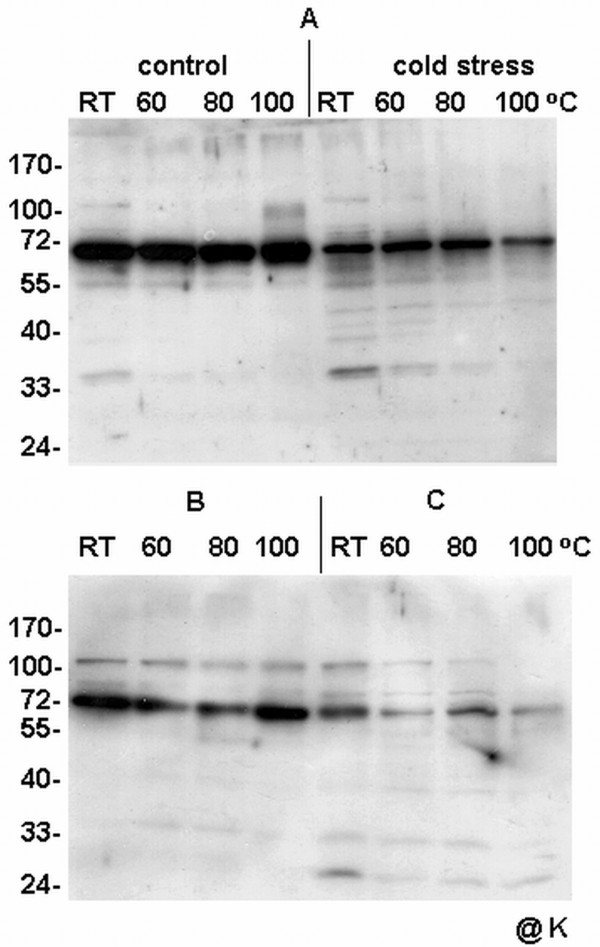
**Analysis of thermostability of mitochondrial dehydrin-like proteins**. The results of the analysis are shown for mitochondrial proteins isolated from yellow lupin hypocotyls (**A**) as well as from cauliflower inflorescences (**B**) and *Arabidopsis *cell cultures (**C**). Lupin mitochondria were isolated from seedlings growing in control conditions and from plants submitted to cold stress (as indicated on panel **A**). The thermostability of dlps was assayed at different temperatures (room temperature- RT, as well as 60, 80 and 100°C). Ten micrograms of mitochondrial proteins were loaded per lane. Western analysis was carried out using the antibody against the K-segment of dehydrins (**@K**). The representative Western blot came from 3 experimental repetitions.

To estimate the thermostability of detected dlps in the mitochondria from cauliflower inflorescences, *Arabidopsis *cell cultures and yellow lupin hypocotyls, a relevant assay was performed. Equal parts of total mitochondrial protein were submitted to various temperatures and analyzed on Western blots using antibodies specific to the K-segment of dehydrins. As shown in Tables 1, 2, 3 and Figure 9, the majority the thermostable proteins belonged to the group of high-molecular weight dlps, above ca. 50 kDa. Most of the dlps below ca. 50 kDa in the mitochondria of the analyzed species remained thermolabile; however, slight variations in the level of thermostability were noticed for dlps from the yellow lupin hypocotyl as well as for those from the *Arabidopsis *cell culture mitochondria (Figure 9). Contrary to the thermostable large (above ca. 70 kDa) dlps from the cauliflower inflorescence mitochondria, the respective proteins from mitochondria of *Arabidopsis *cell culture seem to be significantly more thermolabile (Figure 9, Table 2).

## Discussion

The results of this study indicate the presence of specific mitochondrial dlps in three non-cereal plant species: cauliflower, *Arabidopsis thaliana *and yellow lupin, which considerably extends the results obtained by Borovskii *et al. *[16,24,25]. The detection and partial characterisation of those proteins were performed with the help of different antibodies; two of them were directed against the K-segment of dehydrins and one - against the *Solanum sogarandinum *DHN24 dehydrin (for SK_3_-type of dehydrins). This approach minimized the possibility of detection of artefacts resulting from non-specific cross-immunoreactions. In all the analyses only abundant dlps were taken into account. The two antibodies specific to the dehydrin K-segment gave similar results on all Western blots; however, limited similarity of the dlps pattern was obtained when antibodies against K-segment and against potato DHN24 dehydrin were used. This may be due to the relatively different amounts of K-segments or K-segment- related sequence motifs in different dlps, or to the non-equal number of accessible epitops for dlps recognized by those antibodies. The last possibility is more probable, as in this study the spectrum of dlps that crossreacted with the DHN24 antibody was always smaller. Additionally, stress conditions could affect not only the relative level of dlps, but also the number of immunoreactive epitops of particular proteins. Notably, Rorat *et al. *[6] speculated that the *Brassicaceae *family may be characterized by the presence of two types of cytoplasmic dehydrins: SK_3 _(including DHN24) and SK_2_. Therefore, it cannot be excluded that at least part of the mitochondrial dlps of cauliflower and *Arabidopsis *reported in the present study may contain aminoacid motifs similar to different dehydrins. However, only limited similarity in size of the presently reported cauliflower and *Arabidopsis *mitochondrial dlps with known dehydrins of related plant species was detected, as most of the described *Brassica *dehydrins range from 19 to 22 kDa in size; an exception is the 31 kDa ERD10 dehydrin of *Brassica napus *([GenBank:AAR23753], [28]). The size of ten known *Arabidopsis *dehydrins also varies from 10 to 29 kDa [2,29]. However, RAB18 [Gen Bank:NP_201441, ERD14 [Gen Bank:NP_177745] and LTI30 [Gen Bank:NP_190666] *Arabidopsis *dehydrins as well as 3 other proteins [Gen Bank:NP_195554, NP_195624, NP_179744] resemble in size the dlps detected in *Arabidopsis *mitochondria in the present study. It should be added that despite the fact that all dehydrin subclasses contain K-segment, it may be less conservative in sequence among different plants, as suggested for gymnosperms (reviewed by Close *et al. *[4]). This fact may also indicate that some truly diverse dlps were identified in lupin, cauliflower and *Arabidopsis *mitochondria.

Notably, in cauliflower, *Arabidopsis *and lupin mitochondria two distinct groups of dlps seem to be present: one containing higher-molecular-weight proteins (>50 kDa) and the other composed of small dlps which are more variable in size than in cereal mitochondria (Figures 2, 3, 4, 5; [16]). Previous studies reported tissue specific localisation of plant dehydrins under stress conditions [13]. Here the expression of dlps under the abiotic stress conditions analyzed was also monitored in the mitochondria prepared from different plant organs. This approach made it possible to determine novel dlps in plant species and organs, which extended the existing data. The level of dlps was studied in the mitochondria isolated both from imbibed seeds and hypocotyls of yellow lupin and from cauliflower and *Arabidopsis *leaves as well as from cauliflower early inflorescences. The influence of cold and heat stress on the accumulation of various dlps in the lupin mitochondria from imbibed seeds and hypocotyls was different. Generally, in the lupin imbibed seed mitochondria, contrary to the lupin hypocotyls, the quantitative changes were rather limited to higher-molecular weight dlps; however, the level of some small thermolabile dlps in lupin hypocotyl mitochondria was clearly affected by stress conditions, which was not the case for cereal mitochondria dehydrin-related proteins [16]. In fact, the dlps detected in lupin imbibed seed mitochondria may represent a distinct set of proteins active under dessication, imbibition and germination. However, comparison of dlps of the dried and imbibed seed mitochondria was impossible due to the low quality of the dried seed mitochondria. In addition, it seems that some proteins (ca. 25, 35, 45 kDa) may be good candidates for cold-induced dlps in yellow lupin mitochondria. Moreover, protein 25 kDa, accumulating in yellow lupin hypocotyl mitochondria under heat stress, displayed similarity in size to SK_3_-type RAB16-like *Lupinus albus *dehydrin ([GenBank:AAT06600], [30]); however, yellow lupin 25 kDa protein could not be detected by dehydrin SK_3_-type specific antibodies. This suggests that some lupin mitochondrial dlps may not necessarily be similar to the known dehydrins in the species analyzed up till now.

Significant changes in the abundance of various dlps were also very evident in the mitochondria from *Arabidopsis *leaves and cell cultures. Generally, in the latter case quite numerous dlps were detected (Figure 5), which is consistent with the strong accumulation of SK_2_-type dehydrins in poplar cell cultures in the stationary phase [31]; interestingly, the level of those proteins increased constantly from the early exponential phase. Notably, the *Arabidopsis *cell cultures used in the present study displayed a high number of mitochondrial dlps in the stationary phase of growth. Those dlps were immunorective both with the dehydrin K-segment and with dehydrin SK_3_-type specific antibodies.

The mitochondrial dlps from unstressed cauliflower inflorescences were less abundant, but the level of some of them significantly increased after cessation of cold and heat stress and subsequent plant growth under control conditions. This result can indicate that the changes in dlps abundance in cauliflower mitochondria are time-dependent and associated with the possible acquiring of tolerance both to cold and to heat treatment. It is possible that cytoplasmic dehydrins and dehydrin-related proteins may interact with mitochondrial membranes and prevent them from destabilisation during stress and conditioning to stress. Curiously enough, plant recovery after heat stress, contrary to the direct heat and cold stress in mitochondria of cauliflower inflorescences, resulted in accumulation of dlps quite different in size, possibly because the induction of distinct dlps, specific to heat stress response and acting in protein chaperoning during local water fluctuations. These changes may be present after plant shifting from heat stress to control growth conditions.

Dlps from the mitochondria of cauliflower leaves displayed relatively lower changes in abundance after cold treatment than the *Arabidopsis *ones (Figures 3, 4). Interestingly, Soitamo *et al. *[32] observed similarly sized dehydrins in whole leaf protein extracts from *Arabidopsis *plants under cold/light treatment.

In the present study the most thermostable polypeptides were found among high-molecular weight dlps (Figure 9, Table 1, 2, 3). Generally, the thermostability of mitochondrial dlps described in this study for all the analyzed plants seems not to correlate with their level of accumulation under stress conditions. Yellow lupin thermolabile mitochondrial dlps of ca. 25 kDa displayed differences in the level under cold and heat stress conditions, contrary to the thermolabile 28 kDa protein in cereal mitochondria, which showed constitutive expression [16].

The study of subcellular localisation of cauliflower dlps led to the conclusion that most of those proteins are present in the mitochondrial matrix (Figure 6). Previously Borovskii *et al. *[26] had shown that some small cereal dlps are water-soluble proteins and suggested that other dlps could associate with cellular membranes. The application of pronase E for digestion of native cauliflower mitochondria made it possible to confirm that only some high-molecular weight dlps, like 65 kDa, are sensitive to proteolytic digestion (Figure 8). This corresponds to the results of Borovskii *et al. *[26] in the case of 63 kDa dehydrin-related protein of wheat. The interactions of cauliflower mitochondrial dlps with membranes were also checked during the salt extraction (Figure 7). Generally, the results coming from those experiments were fully consistent with those obtained from pronase digestion. The cauliflower dlp of ca. 65 kDa (probably corresponding to wheat 63 kDa protein) was removed from the membranes, which suggests its rather peripheral localisation on the external side of the outer mitochondrial membrane (Figure 8). Considering the high level of thermostability as well as the results coming from salt extraction and pronase E digestion, the protein of ca. 65 kDa seems to be a putative candidate for a true cauliflower cytoplasmic dehydrin interacting with mitochondria. The possibility of interactions of dehydrins and dehydrin-related proteins with higher plant mitochondria should be confirmed in future by functional targeting assays, unless the cloning of cDNAs for those proteins becomes possible.

The results of the current study made it possible to discuss again [16] the putative roles of dehydrins and dehydrin-related proteins in plant mitochondria under abiotic stress treatment. It seems that some large thermostable putatively cytoplasmic dehydrins associate with the outer mitochondrial membrane, whereas smaller dehydrin-related proteins are actively imported into the mitochondrial matrix and therefore could also stabilize the inner mitochondrial membrane and/or protect matrix enzymes against damage. During acclimation to stress conditions, the interactions of cytoplasmic dehydrins with the outer mitochondrial membrane may get strengthened and in the mitochondrial matrix the pool of dehydrin-like proteins may be actively rearranged in number and size. This phenomenon indicates that some conserved mechanisms regarding the expression of genes encoding mitochondrial dehydrin-related proteins may be engaged in stress response. This observation should be additionally confirmed by functional tests in the future.

## Conclusions

A broad spectrum of dehydrin-like proteins accumulating in response to cold and heat stress in mitochondria of three non-cereal, higher plant species (cauliflower, *Arabidopsis *and yellow lupin) was shown. The proteins detected in the present study were considered 'dehydrin-like', on the basis of the immunoaffinity with the dehydrin K-segment, with the dehydrin SK_3 _motif, and also partly because of their thermolability. They display limited similarity in size to those reported previously in maize, wheat and rye mitochondria. Changes in the level of these proteins, in the cauliflower inflorescences, were more evident under acclimation to stress. The pool of dlps from different organs of the analyzed plants generally varied. In most cases, some small dehydrin-like proteins were accumulated under the stress conditions used and therefore they are likely to be involved in stress response. Most of the mitochondrial dehydrin-like proteins from cauliflower inflorescences were found probably matrix localized; nevertheless, a putative candidate (65 kDa) for a membrane-associated one was also proposed. Therefore, plant mitochondrial dlps can be involved both in the protection of important soluble enzymes and/or in the control of the physicochemical status of mitochondrial membranes.

## Methods

All the experimental procedures used in this study were conducted three times.

### Plant material and growth conditions

The seeds of yellow lupin (*Lupinus luteus *cv. Topaz) were provided by the Plant Breeding Station (Wiatrowo, Poland). Prior to imbibition, the seeds were surface-sterilized in 0.7% (w/v) calcium hypochlorite for 15 min and extensively washed with sterile water. Afterwards, the lupin plants were grown in darkness at 23°C for 6 days, on sterile vermiculite. The seeds of cauliflower (*Brassica oleracea *var. *botrytis *cv. Diadom) were provided by Bejo Zaden Poland. The cauliflower plants were grown at 23/19°C (day/night) under photon flux density 200 μmol · m^-2 ^· s^-2 ^(16 h fotoperiod in the phytotron Versatile Environmental Test Chamber MLR-350, Sanyo) for 30 days or grown in the cultivation chambers at the local breeding station (University of Life Sciences, Poznan, Poland) under the same conditions for 2 months. For the induction of inflorescences, the plants were kept at a temperature lowered to 15°C for two weeks; then the temperature was elevated to 23°C in the third month. The seeds of *Arabidopsis thaliana *cv. Columbia were surface-sterilized with 70% ethanol for 10 minutes and washed with 95% ethanol. The *Arabidopsis *plants were grown for 30 days in the phytotron under the same conditions as those described above for the cauliflower. An *A. thaliana *cv. Landsberg suspension culture was maintained as described by Uyttewaal *et al. *[33].

### Stress treatment and tissue collection

After imbibition the lupin seeds were transferred onto sterile Whatman 3 MM paper wetted with sterile water, and incubated for 12 h at 23°C and then for the next 12 h at 8°C (cold treatment). In the case of heat treatment, the seeds were placed onto Whatman paper for 22 h at 23°C and then transferred to 40°C for 2 h. Before the isolation of mitochondria, the seed cover was removed. Lupin hypocotyls were collected from 6-day-old seedlings. For the application of cold stress, lupin seedlings grown in vermiculite were transferred to 8°C for 72 h before the isolation. Heat stress (40°C) was applied for 2 h before the isolation of lupin seedlings. The cauliflower leaves and *Arabidopsis *rosette leaves were collected from 1-month-old plants. For the application of cold stress, the plants - before the isolation of mitochondria - were transferred for 10 days to 8°C. Heat treatment (40°C) was applied to the growing plants for 4 h before the isolation of mitochondria. Frost treatment (-20°C) was applied to the growing plants for 30 min until the moment of appearance of ice on the surface of the leaves. Cauliflower inflorescences (5 mm topmost layer, floral heads of 10-15 cm in diameter) were collected from 3-month-old plants. The cold and heat stress treatment applied to the cauliflower plants with inflorescences was the same as in the case of the collected cauliflower leaves. After stopping the stress treatment, part of the cauliflower plants were transferred to the standard growth conditions for 48 h. The inflorescences were harvested either directly after stopping the stress treatment or after stopping the additional cultivation of plants under control conditions.

### Isolation of mitochondria

In all the cases, the homogenisation buffer used for the isolation of mitochondria contained the Plant Protease Inhibitor Coctail (Sigma). The mitochondria from the lupin seeds were isolated using differential centrifugation and Percoll separation of crude organelles [34]. The homogenisation buffer contained 0.5 M sucrose, 1 mM EDTA, 2.5 mM MgCl_2_, 50 mM potassium phosphate buffer (pH 8.0), 30 mM sodium ascorbate, 2% (w/v) PVP-40 and the uniform gradient of 28% Percoll was applied for the obtaining of pure mitochondria. The mitochondria from the lupin hypocotyls were isolated as described by Karpinska and Augustyniak [35] - without DNase I treatment and using the 3-step Percoll gradient [36]. The mitochondria from the cauliflower inflorescences were isolated as described by Pawlowski *et al. *[37]. The mitochondria from the cauliflower and *Arabidopsis *leaves were isolated using the protocol of Werhahn *et al. *[38] with modifications according to Kruft *et al. *[39]. The mitochondria from *Arabidopsis *suspension cultures were isolated as described by Giegé *et al. *[40]. During the resuspension of all the mitochondria in washing buffer, the Complete Mini EDTA-free Protease Inhibitor Coctail (Roche) was added. Purity assays of isolated mitochondria (measurement of activities of mitochondrial cyt. *c *oxidase, peroxysomal catalase, plastid alkaline pyrophosphatase and cytoplasmic alcohol dehydrogenase) were conducted according to Pawlowski *et al. *[37]. Besides this, the purity of isolated mitochondria was verified by transmission electron microscopy (JEOL 1200EXII, Jeol).

### Preparation of submitochondrial fractions

The mitochondria suspended in washing buffer (0.4 M mannitol, 10 mM potassium phosphate buffer, pH 7.2, containing also the Complete Mini EDTA-free Protease Inhibitor Coctail (Roche) were diluted with 10 mM potassium phosphate buffer (pH 7.2), broken by three cycles of freezing/thawing in liquid nitrogen, and sonicated 4 times for 10 s using an ultrasonic desintegrator UD-11 (Techpan). Unbroken mitochondria were removed by centrifugation at 5000 g for 10 min at 4°C, and a supernatant containing mitochondrial lysate was used for further fractionation. A fraction enriched in mitochondrial membranes was obtained by centrifugation of mitochondrial lysate at 100000 g (angle rotor 70.1 Ti, L8-60 M Beckman ultracentrifuge) for 1 h at 4°C. The pellet was suspended in washing buffer containing also the Complete Mini EDTA-free Protease Inhibitor Coctail (Roche). The supernatant represented a fraction enriched in mitochondrial matrix soluble proteins.

### Isolation of peripheral proteins

In order to obtain peripheral proteins, fractions enriched in mitochondrial membranes were treated with increased concentrations of KCl (0.25, 0.5 and 0.75 M). Peripheral proteins were also extracted by alkali treatment (100 mM Na_2_CO_3_, pH 11.5) for 30 min at 4°C. After centrifugation at 100000 g (angle rotor 70.1 Ti, L8-60 M Beckman ultracentrifuge) for 10 min at 4°C, peripheral membrane proteins were in the supernatant and integral proteins remained in the pellet. Integral and peripheral protein fractions were resuspended in PBS, cold acetone precipitated, centrifuged and resuspended in washing buffer containing also the Complete Mini EDTA-free Protease Inhibitor Coctail (Roche).

### Thermostability tests

Equal amounts (10-20 μg) of mitochondrial proteins suspended in washing buffer, containing also the Complete Mini EDTA-free Protease Inhibitor Coctail (Roche), were incubated 15 min on ice, or at 60, 80 or 100°C and immediately transferred to ice for 15 min. After centrifugation (15000 g in 5 min at 4°C), thermostable proteins present in the supernatants were denatured for 3 min at 95°C and directly loaded onto SDS polyacrylamide gel.

### Pronase E treatment

For the assay, 30 μg of freshly isolated mitochondria suspended in washing buffer without any protease inhibitor and 20 μg of pronase E (Serva) were used. In the control reaction, pronase was replaced by washing buffer. Proteolytic digestion proceeded at 30°C or 37°C at three time intervals (10 min, 30 min, 1 h). The reaction was stopped by the immediate transfer of the reaction mixture on ice and addition of the Complete Mini EDTA-free Protease Inhibitor Coctail (Roche). Mitochondria were then diluted ten times with washing buffer and samples were centrifuged at 15000 g for 5 min at 4°C. The pellets containing mitochondrial proteins were suspended in 20 μl of washing buffer containing 1 mM PMSF (Sigma) and directly loaded onto SDS polyacrylamide gel.

### Western blot analysis

The protein content was determined by the BioRad Protein Assay. Proteins were separated on SDS-PAGE and electroblotted onto polyvinylidene difluoride Immobilon-P membranes (Millipore), using a semidry blotting apparatus. The membranes were stained with Commassie Brilliant Blue to ensure that equal amounts of proteins were transferred. After completion of the destaining and the subsequent blocking of the membrane, they were incubated overnight with rabbit polyclonal antibodies. Three kinds of primary dehydrin antibodies were used: 1) the antibody immunoreacting with the K-segment of dehydrin (dilution 1:1000, [27]); 2) the antibody raised against the K-segment of dehydrins, with N terminal cysteine on the synthetic peptide (dilution 1: 1400, Stressgen); 3) the antibody against the *Solanum sogarandinum *DHN24 dehydrin (dilution 1: 500, [6]). Control immunoassays were also performed with the dehydrin K-segment specific antibody [27] blocked with the synthetic peptide TGEKKGIMDKIKEKLPGQH [27] (with the 100-fold molar excess of the peptide to IgG in the serum). For the monitoring of the quality of mitochondria during assays, the following control antibodies were used: 1) the antibody against NAD9 subunit of wheat complex I (dilution 1: 50000, [41]); 2) the yeast cyt. *c*_1 _antibody (dilution 1: 20000); 3) the yeast cyt. *c *antibody (dilution 1:5000); 4) the *Arabidopsis *SHMT antibody (dilution 1:5000, Agrisera); 5) the *Arabidopsis *VDAC-1 antibody (dilution 1:1000, Agrisera); 6) the *Arabidopsis *CCMH antibody (dilution 1:500, [42]). Bound antibodies were detected using an anti-rabbit immunoglobulin G horseradish peroxidase conjugate diluted 1:10000 (BioRad) and visualized with enhanced chemiluminescent reagents (GE Healthcare). Western blot images in triplicate were analyzed by Multi Gauge (v. 2.2) software and the representative pattern was presented. Western blot band intensities were calibrated to the protein loading in the linear relationship (the 2-fold protein amount resulted in the 2-fold difference in Western blot band relative intensity).

## List of abbreviations

ABA: Abscisic acid; CCMH: Protein of cytochrome *c *maturation pathway; COR: Cold-responsive; Cyt.: Cytochrome; DHN24: Dehydrin of 24 kDa from *Solanum sogarandinum *(containing a single S-segment and three K-segments); Dlp(s): Dehydrin- like protein(s); ERD: Early responsive to dehydration; IDH: Isocitrate dehydrogenase; K-type dehydrin: Dehydrin containing K-segment; LEA: Late embryogenesis abundant; LTI: Low temperature induced; NAD9: Subunit of mitochondrial complex I; RAB: Abscisic acid-responsive; SDS-PAGE: SDS polyacrylamide gel electrophoresis; SHMT: Serine hydroxymethyltransferase; SK_n_-type of dehydrins: Dehydrins containing a single S-segment and n K-segments; VDAC-1: Isoform 1 of voltage dependent anion channel

## Authors' contributions

MR conceived the study and performed the experiments, analyzed the images and wrote the manuscript. Author read and approved the final manuscript.
